# Left ventricular ischemia after arterial switch procedure: Role of myocardial perfusion scintigraphy and cardiac CT

**DOI:** 10.1007/s12350-019-01738-4

**Published:** 2019-05-22

**Authors:** Marie Louise E. Bernsen, Josephina C. C. Koppes, Bart Straver, Hein J. Verberne

**Affiliations:** 1 Department of Radiology and Nuclear Medicine, Amsterdam University Medical Center, Location AMC, University of Amsterdam, Meibergdreef 9, 1109 AZ Amsterdam, The Netherlands; 2 Department of Cardiology, Amsterdam University Medical Center, Location AMC, University of Amsterdam, Amsterdam, The Netherlands

**Keywords:** Myocardial ischemia and infarction, CT, SPECT, Gated SPECT

## Abstract

Transposition of the great arteries is a congenital heart defect defined by an abnormal connection between the aorta, pulmonary artery, and the ventricles, resulting in parallel systemic and pulmonary circulations. Long-term follow-up data of patients who underwent correction via an arterial switch operation have recently shown that as a result of re-implantation of the coronary arteries in the neo-aorta, coronary stenosis and occlusion are relatively common complications. In this report, we discuss two cases illustrating the added value of myocardial perfusion imaging (MPI) and cardiac CT for the assessment of these patients. Based on the available literature we conclude that MPI and cardiac CT are excellent non-invasive methods to evaluate coronary anatomy and myocardial function also in this specific group of patients.

**Electronic supplementary material:**

The online version of this article (10.1007/s12350-019-01738-4) contains supplementary material, which is available to authorized users.

## Introduction

Transposition of the great arteries (TGA) is a congenital heart defect with an incidence of around 2.3 in 10.000 live births.^1^ In this condition, the aorta is connected to the morphological right ventricle and the pulmonary artery arises from the morphologic left ventricle. This results in separate and most often independent parallel systemic and pulmonary circulations. Usually the aorta lies right and ventral of the pulmonary artery and the position of the right ventricle is on the right side, so-called d(extro)-transposition.^2^ In 70% of the cases, this condition is isolated; in 30%, there is a complex form where the transposition is associated with a relevant ventricular septum defect (VSD), an obstruction of the left ventricular outflow tract (LVOT), or a combination of both.

As a result of this transposition, the systemic and pulmonary circulations run in parallel. The venous blood is re-introduced in to the systemic circulation, without crossing the pulmonary circulation for oxygenation. Because of the separate pulmonary and systemic circulation, children present with severe arterial desaturation at birth. To support life there has to be a connection between the 2 circulations, either via an atrial septal defect (ASD), a VSD, or patent arterial duct. The neonate oxygen saturation of the systemic circulation is completely dependent on shunting through these connections.

When TGA is diagnosed, prostaglandins are used to prevent the arterial duct from closing. In addition, the resistance in the pulmonary vascular bed is lowered after birth. This results in an increased left-right shunt and thus improved pulmonary flow. In turn this leads to increased volume and pressure in the left atrium and thus left-right shunt on atrial level (given an ASD or VSD), creating an increased and oxygen-rich blood inflow from the left atrium and as a result higher oxygen saturation in the systemic circulation.

Surgical treatment of TGA is one of the most successful achievements in the history of congenital heart defects surgery. Without treatment, 30% of the children die within 1 week after birth, 50% in one month, and 90% within one year. After surgical repair, mortality and morbidity rates decrease dramatically.^3^

First step in treatment is stabilization by creating a shunt possibility on the atrial level, usually through balloon septostomy (so-called Rashkind procedure).^4^ This procedure is performed in the first weeks after birth, followed by corrective surgery. There are several surgical repair options; however, the most used method nowadays is an anatomical correction through the arterial switch procedure. This procedure restores the normal anatomic form of the circulation. The aorta and pulmonary artery are transected and switched. Furthermore, there is detachment of the coronary arteries along with a “button” from the aortic wall, followed by a re-implantation in the neo-aorta.^5^ Main advantage of this procedure is that the role of the left ventricle as systemic ventricle is maintained.^2^

In 5%-15%, long-term complications occur; the most important being pulmonary hypertension caused by stenosis of the pulmonary artery or in other levels of the right ventricular outflow tract (RVOT).^6^ In addition, the patency and function of the re-implanted coronary arteries are reason for concern.^7^–^9^ Other complications are related to the neo-aorta/ aortic valve. 10

## Case presentation

We present two cases of teenage patients with a history of transposition of the great vessels and arterial switch procedure.

### Case 1

A 13-year-old boy presented with signs of ischemia on ECG during exercise stress test. Exercise testing was performed according to the Bruce protocol. ST-segment changes were observed after 12-15 minutes of exercise. There was ST depression in leads V4-V6 and II, aVF. The patient had no clinical complaints and exercise test was performed on routine clinical evaluation.

**Figure 1:**
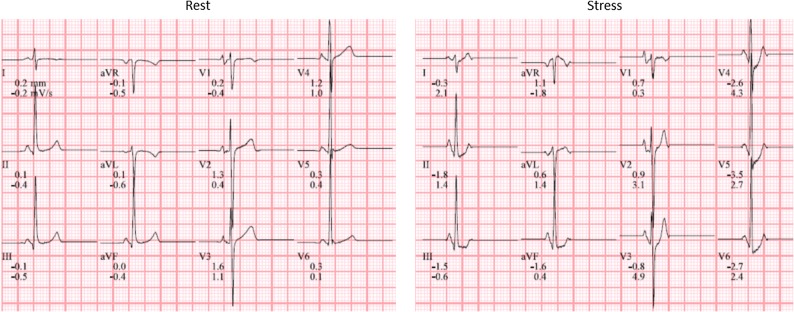
Rest electrocardiogram (left) showing sinus rhythm and no signs of ischemia. Stress ECG (right) with significant ST-segment depression (up-sloping) in leads V4-V6

Gated single-photon emission computerized tomography (SPECT) myocardial perfusion imaging (MPI) after symptom limited bicycle ergometry and at rest was performed to rule out myocardial ischemia (2-day stress rest protocol). Patient exercised for 9 minutes with a stress/exercise tolerance of 123 Watt and a maximum heart rate of 181 beats/minute (87% of the age-predicted maximum heart rate). Rest ECG showed no abnormalities. On stress ECG ST depression was seen at the end of the exercise (V4-V6), with normalization during recovery (Figure 1). Administered dose was 434 MBq ^99m^Tc-tetrofosmin (stress) and 487 MBq ^99m^Tc-tetrofosmin (rest).

**Figure 2:**
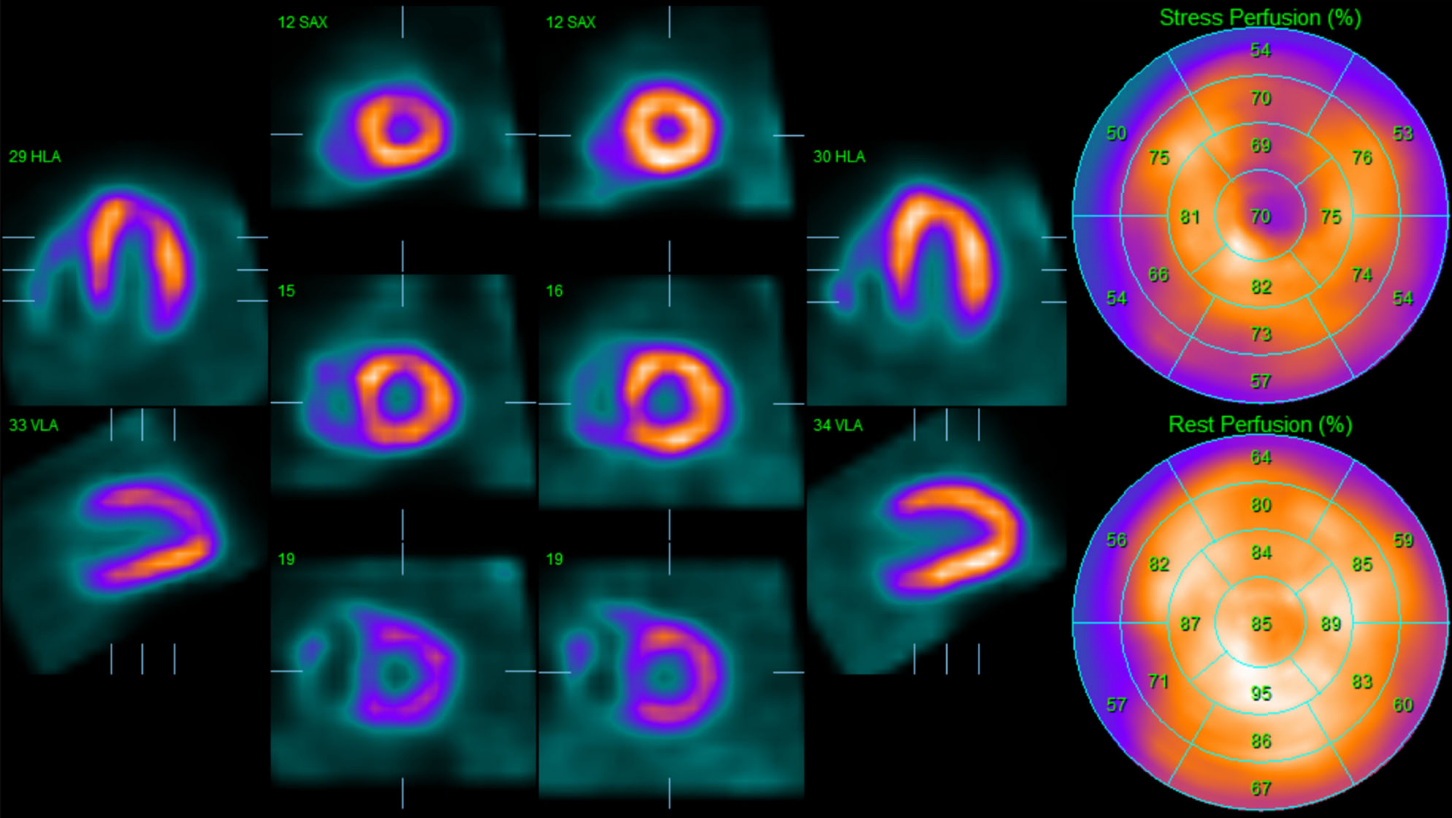
^99m^Tc-tetrofosmin stress/rest myocardial perfusion imaging at initial screening. During stress there is a marked perfusion defect in the anterior wall (segment 1, 7, 13, 17), with complete normalization of myocardial perfusion at rest

MPI showed a non-dilated left ventricle with a large area of inducible hypoperfusion in the anterior wall, running from the left ventricular base till the apical segments (4 segments), approximately involving 10% of the left ventricle (Figure 2). There was a minor but most likely significant decline in systolic function after stress (EF = 68 % vs. 74%).

**Figure 3:**
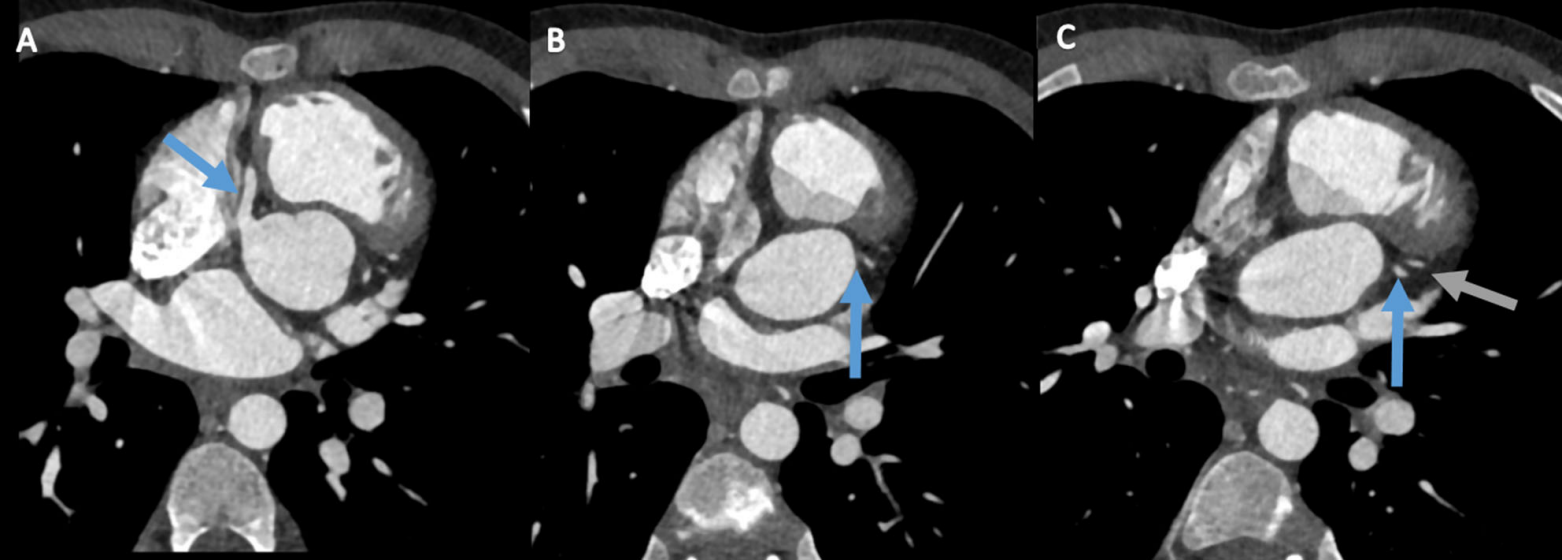
Cardiac CTA displaying a normal course and filling of the RCA (**A**, blue arrow). There is a stenotic origin of the LCA (**B**, blue arrow). More distal there is filling of the LAD (**C**, gray arrow) and Cx (**C**, blue arrow)

**Figure 4:**
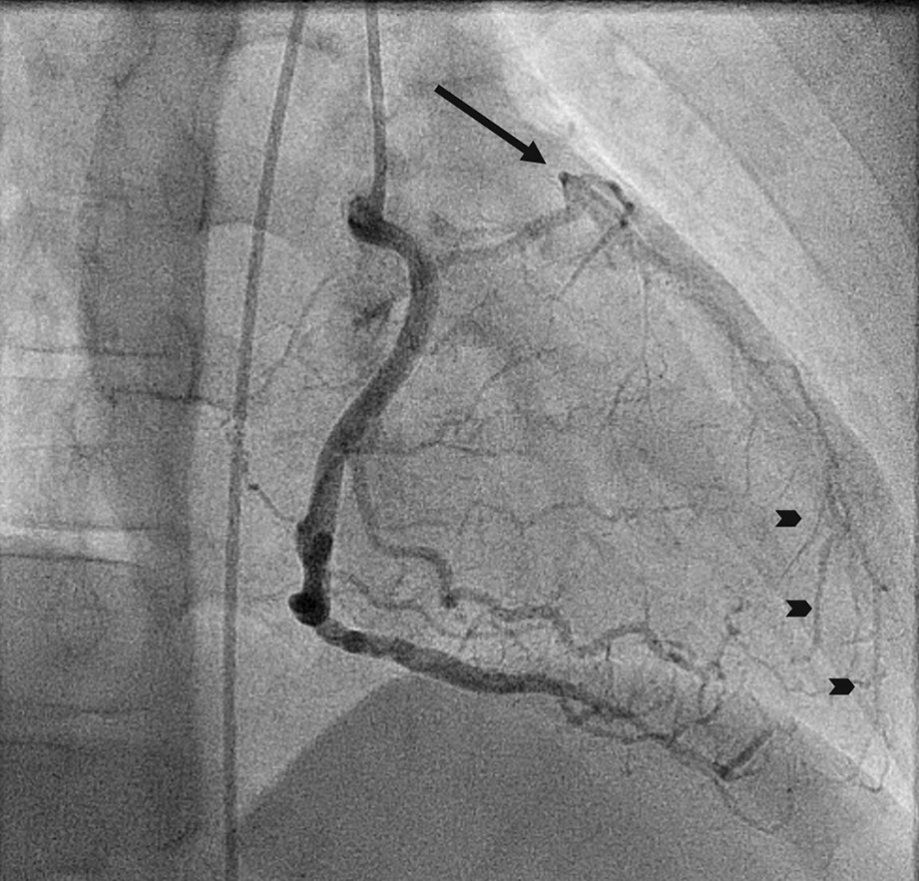
Coronary angiography showing a monocoronary artery (RCA) with post-switch occluded LCA (arrow). There is retrograde filling of the LCA via collateral circulation (arrowheads)

Contrast-enhanced cardiac CT showed a normal aspect and course of the RCA. There was an occlusion at the origin of the LCA. More distal there was filling of the LAD and Cx, probably by collaterals and retrograde filling via the RCA (Figure 3). Coronary angiography (CAG) confirmed CT findings (Figure 4).

**Figure 5:**
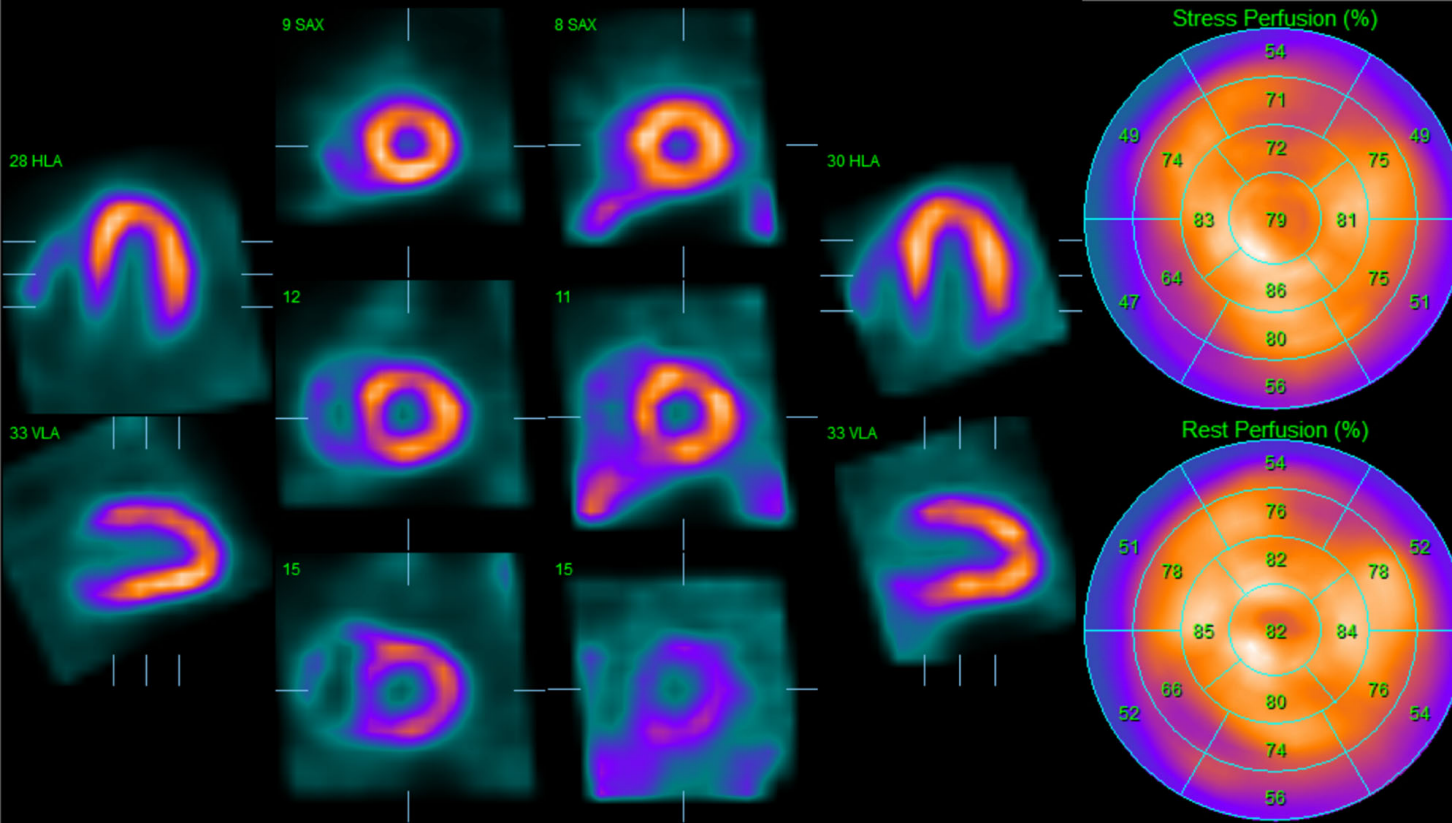
^99m^Tc-tetrofosmin stress/rest myocardial perfusion imaging 3 months after CABG. Apart from a small area of reversible perfusion abnormality mid-anterior (segment 7), there is a normalized myocardial perfusion

After multidisciplinary consultation a CABG was performed (LIMA-LAD). MPI was repeated after CABG and showed significant improvement of myocardial perfusion, with only a small area of inducible hypoperfusion remaining in the mid-anterior wall (1 segment, SD 0 %) (Figure 3). There was no significant difference between systolic function during stress and rest (EF = 70 % vs. 69%) (Figure 5).

### Case 2

A 15-year-old boy with a history of arterial switch for TGA presented with reduced exercise tolerance.

**Figure 6:**
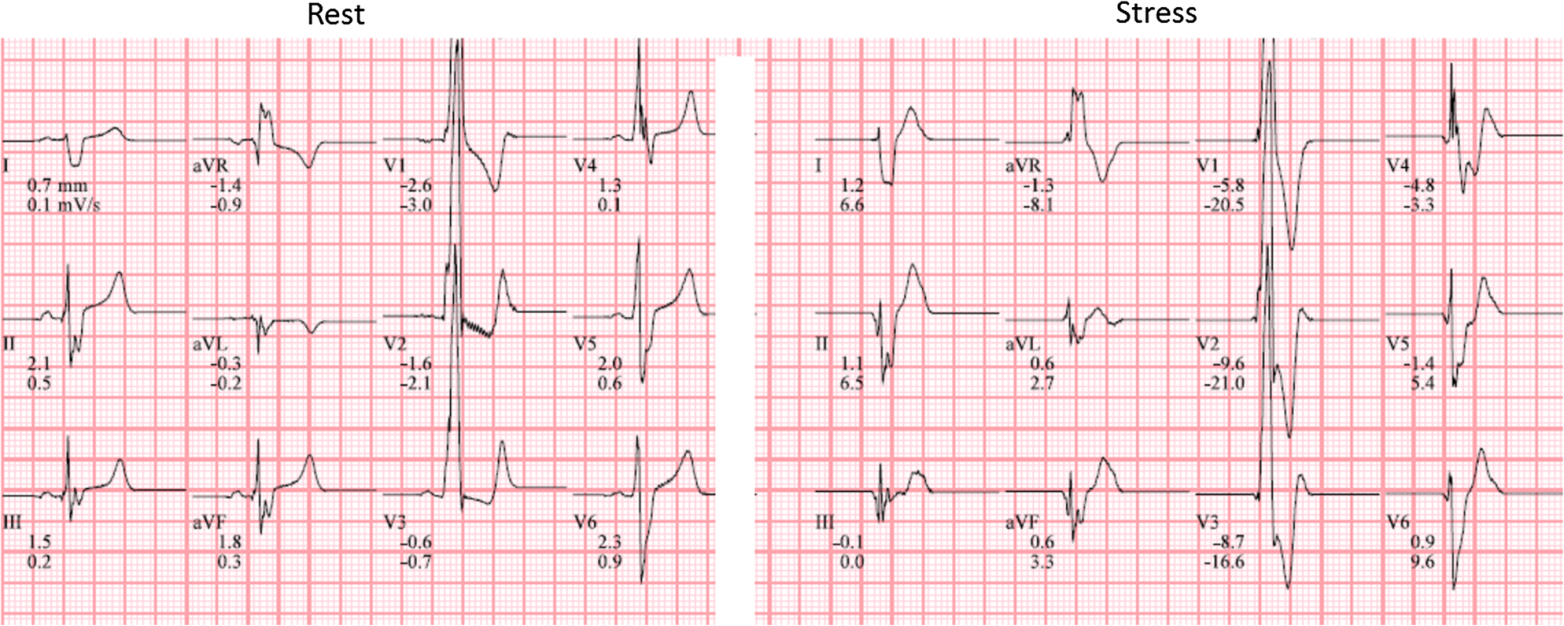
Rest electrocardiogram (left); sinus rhythm with indifferent cardiac axis and a complete right bundle branch block. High amplitudes in the right precordial leads suggesting right ventricular hypertrophy. During stress (right) no significant electrocardiographic changes were seen

^99m^Tc-tetrofosmin stress/rest myocardial perfusion was performed to rule out ischemia (2-day stress rest protocol). Patient exercised during 11 minutes with a stress/exercise tolerance of 87 Watt and a maximal heart rate of 150 beats/minute (73% of the age-predicted maximum heart rate). Exercise was discontinued because of recognizable dyspnea complaints. Rest ECG showed a sinus rhythm with indifferent cardiac axis and a complete right bundle branch block. There were high amplitudes in the right precordial leads indicating a right ventricle hypertrophy. There were no evident signs of ischemia on stress ECG (Figure 6). Administered dose at stress and rest was 385 and 405 MBq ^99m^Tc-tetrofosmin, respectively.

**Figure 7:**
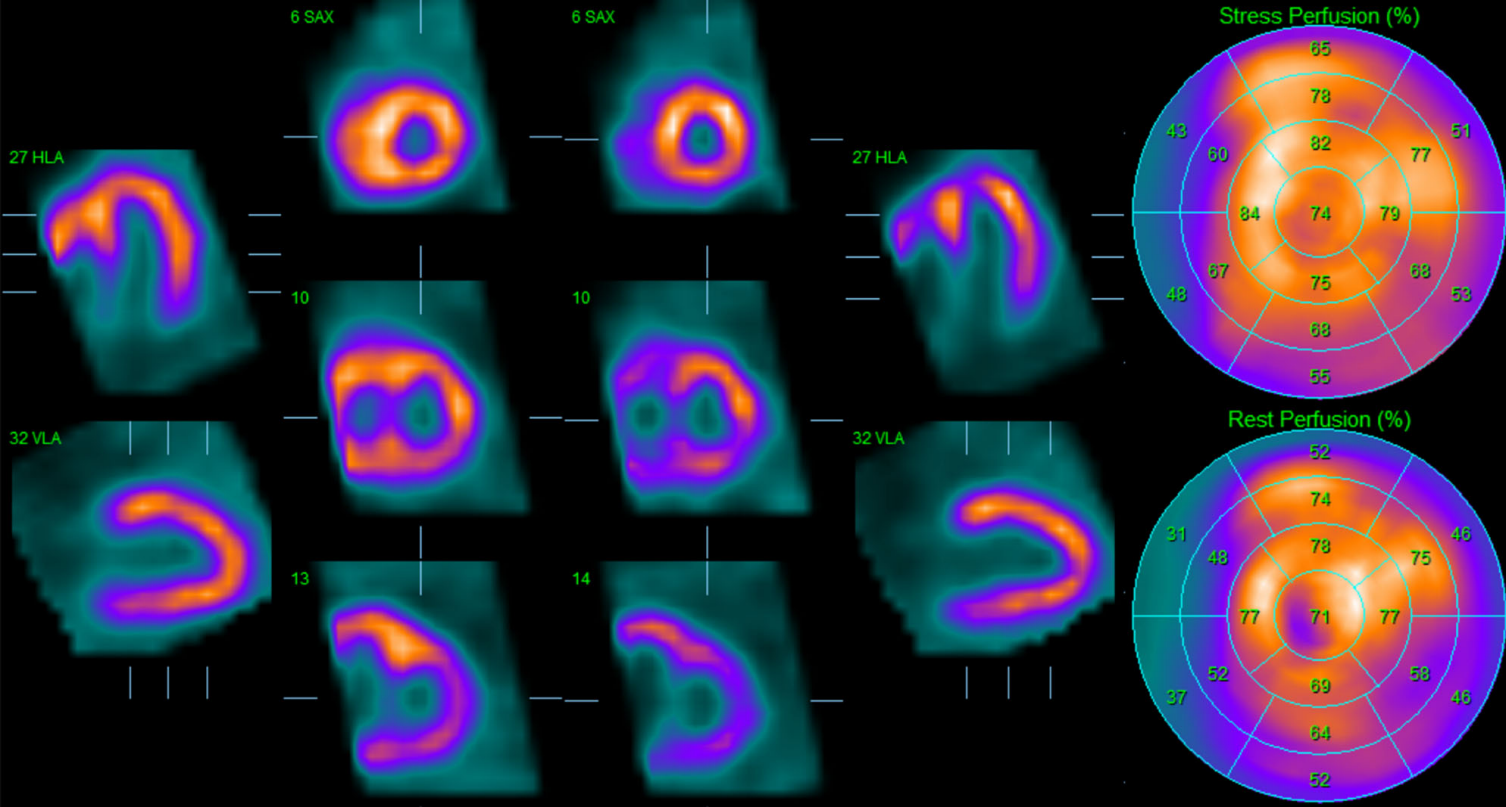
^99m^Tc-tetrofosmin stress/rest myocardial perfusion imaging showed predominant right ventricle wall visualization on post-stress imaging with normalization of myocardial perfusion at rest

MPI showed predominant right ventricle wall visualization on post-stress imaging (Figure 7). During rest, uptake in the left ventricle normalized and right ventricle was less visualized resulting in a normal perfusion ratio between left and right ventricle. There was no difference in systolic function between stress and rest.

**Figure 8:**
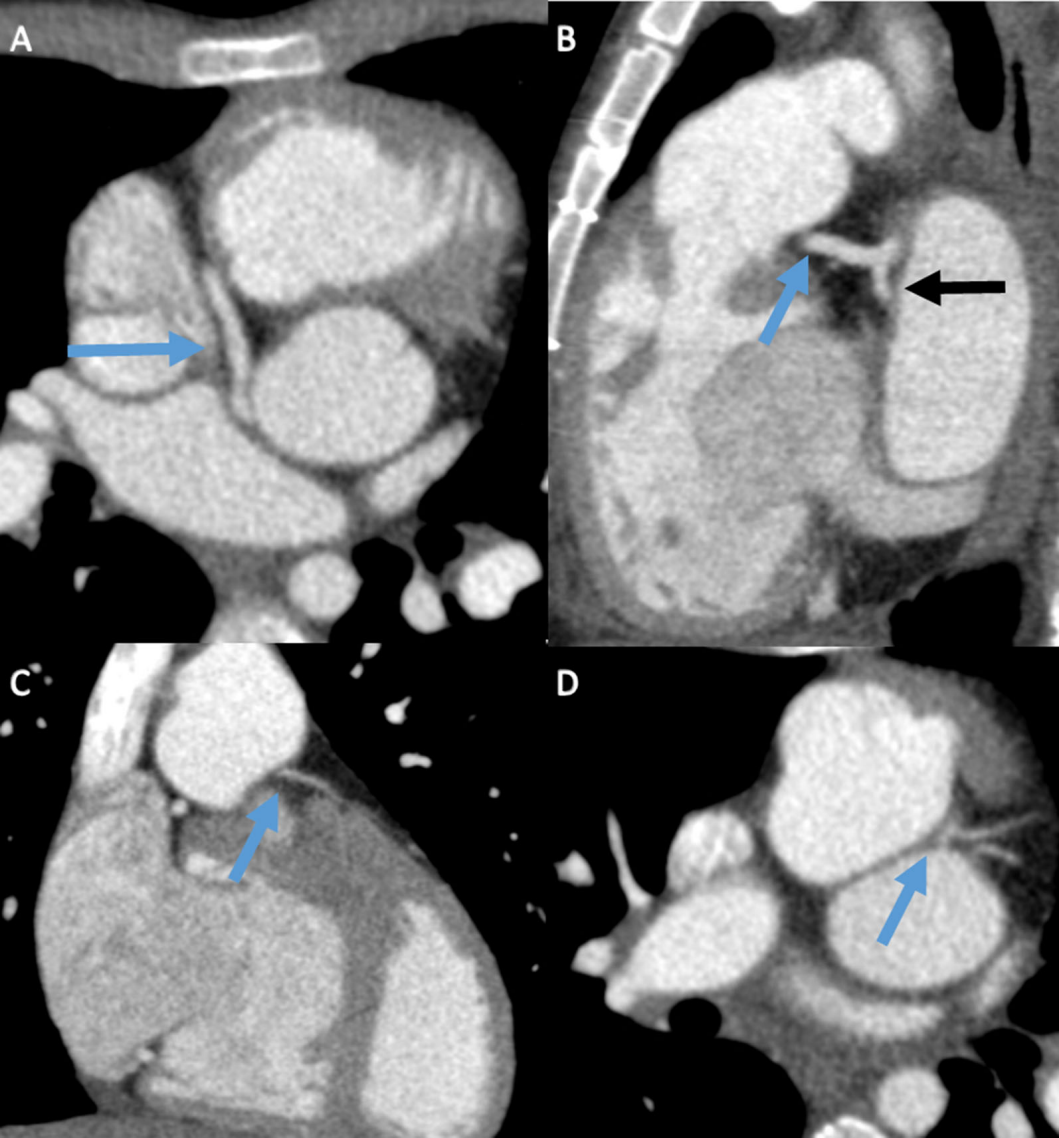
Cardiac CTA demonstrates filling of the RCA (**A**, blue arrow), an aberrant origin of the Cx arising from the RCA (**B**, black arrow). There is a fragile LAD (blue arrow) with suggestion of severe origin stenosis on coronal (**C**) and axial views (**D**)

CT showed a common coronary variant with an aberrant Cx arising from the RCA. There was a severe stenosis/occlusion at the origin of the re-implanted LAD (Figure 8).

**Figure 9:**
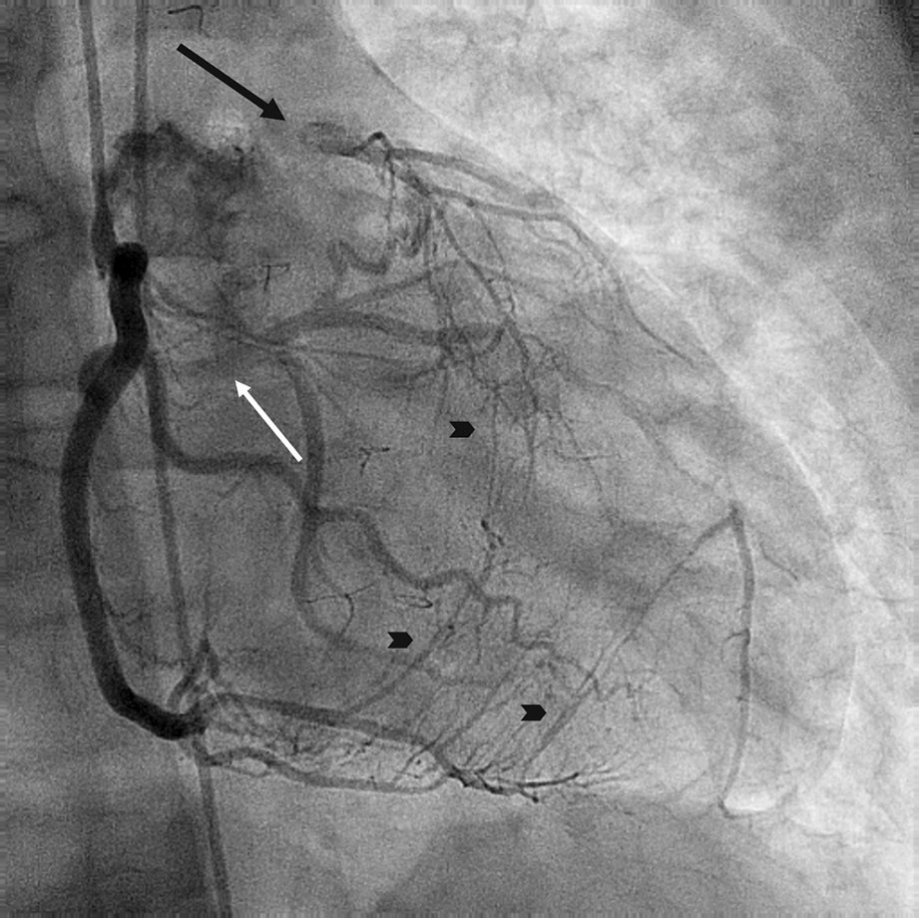
Coronary angiography showing an aberrant Cx arising from the RCA (white arrow). There is a total occlusion of the proximal LAD (black arrow) with evident retrograde filling via collateral circulation (arrowheads)

CAG showed in addition that there was retrograde filling of the LAD trough collateral vessels arising from the RCA (Figure 9).

After multidisciplinary consultation a less aggressive approach was chosen (optimizing anti-ischemic medication for now). This patient is still in follow-up and doing fine (6-month follow-up).

## Discussion

Coronary artery complications are relatively common after an arterial switch procedure. In a study of Bonnet et al., evaluating 64 patients who underwent an arterial switch procedure, 12% of the patients had stenosis or occlusion of the coronary arteries on long-term follow-up (i.e., 7 years).^8^ Complex native coronary anatomy was associated with the occurrence of late coronary artery stenosis, while the re-implantation technique had little-to-no impact on coronary artery prognosis.^7^

In young childhood, these coronary artery lesions do not (always) cause ischemia because the formation of an extensive collateral network maintains left ventricular blood supply. As patients grow older, collateral network may fall short, resulting in ischemia.

In our patients, the presence of a significant collateral circulation from the RCA to the left coronary arterial system maintained perfusion in LAD territory at rest. At stress however, the collateral network failed, causing myocardial ischemia. 11 In addition, in one of the patients the only sign of ischemia on MPI was a predominant RV uptake at stress with a normalized perfusion at rest. In general, visualization of the right ventricle post-stress on MPI is considered a poor prognostic sign and indirect evidence of severe left-sided coronary artery disease/global ischemia (the so-called flip-flop phenomenon).^12^–^14^

All individuals with congenital heart disease (CHD) require some sort of follow-up to monitor cardiac function, disease progression, and the occurrence of complications whether or not related to surgical correction. Transthoracic echocardiography (TTE) is, because of its widespread availability and low cost, the mainstay for follow-up for most CHD. TTE has several limitations, mostly related to limited field of view, which may inhibit the visualization of essential structures such as the great vessels. Usually cardiac MRI is the first choice when information from TTE is incomplete, especially in younger patients. However, when small structures such as coronary arteries are the subject of investigation contrast-enhanced cardiac CT is a better option because of its superior spatial resolution and fast acquisition time.^15^

Early studies opted that non-invasive screening tools for coronary artery insufficiencies in patients with an arterial switch procedure were inadequate because of relatively high radiation doses and low sensitivity and that screening should be performed by invasive coronary angiography.^7^,^16^,^17^ However, with recent developments in CT technology, resulting in improved spatial and temporal resolution as well as multiple techniques on dose reduction and prospective ECG-triggered scanning, the coronary artery anatomy in children can be visualized without the need of invasive coronary angiography.^18^ Similar developments on dose reduction have also taken place in MPI, mostly by improvement of the technology in gamma camera’s resulting in improved count sensitivity for the detection of gamma rays.^19^ To gain information about perfusion, cardiac MRI and nuclear techniques such as scintigraphy and PET imaging are essential. In the current guidelines for patients with a history of arterial switch operation, it is recommend to undergo ischemia testing every 3-5 years, but there is no consensus on what sort of imaging test should be used. As a result a combination of tests such as MRI, CTA, MPI, exercise testing is performed.^15^,^20^

In favor of using MPI in these patients is the known relation between the outcome of exercise MPI and cardiovascular risk. 21 In addition, the guidelines recommend angiography at least once during adulthood to assess coronary patency.

## New Knowledge Gained

In patients with complaints or new ECG abnormalities after an arterial switch procedure, MPI and cardiac CT are essential in the functional and anatomical evaluation of coronary arteries. Furthermore, it is important to realize that prominent visualization of the right ventricle on post-stress myocardial perfusion scintigraphy as the only sign of diffuse left ventricular ischemia also occurs in children and young adults.

## Conclusion

Late outcome of the arterial switch procedure in TGA may be influenced by complications concerning the coronary arteries. Complete occlusion may occur early in life and is often counterbalanced by extensive collateral perfusion. As patients grow older, this collateral network may fall short, resulting in (diffuse) ischemia of the left ventricle. With recent CT developments, especially on dose reduction, CT and myocardial perfusion scintigraphy (MPS) are excellent non-invasive methods to assess coronary anatomy and function. Furthermore, it is essential to be aware that prominent visualization of the right ventricle on post-stress MPS may be the only sign of diffuse left ventricular ischemia (i.e., flip-flop phenomenon).

## Electronic supplementary material

Below is the link to the electronic supplementary material.

Supplementary material 1 (MP4 9437 kb)

Supplementary material 2 (MP4 12016 kb)

## Abbreviations

TGA: Transposition of the great arteries
VSD: Ventricle septum defect
ASD: Atrial septum defect
MPI: Myocardial perfusion imaging
CAG: Coronary angiography
CABG: Coronary artery bypass grafting
LCA: Left coronary artery
LAD: Left anterior descending coronary artery
RCA: Right coronary artery
Cx: Circumflex artery

## References

[1] Reller MD, Strickland MJ, Riehle-Colarusso T, Mahle WT, Correa A (2008). Prevalence of congenital heart defects in metropolitan Atlanta, 1998-2005. J Pediatr..

[2] Warnes CA (2006). Transposition of the great arteries. Circulation..

[3] Bouma BJ, Meijboom FJ, Mulder BJ. Transpositie van de grote arterien. In: Mulder BJ, Pieper PG, Meijboom FJ, Hamer JP, editors. Aangeboren hartafwijkingen bij volwassenen. Bohn Stafleu van Loghum; 2013. p. 136.

[4] Rashkind WJ, Miller WW (1966). Creation of an atrial septal defect without thoracotomy. JAMA..

[5] Marathe S, Talwar S (2015). Surgery for transposition of great arteries: A historical perspective. Ann Pediatr Cardiol..

[6] Jenkins KJ, Hanley FL, Colan SD, Mayer JE, Castañeda AR, Wernovsky G (1991). Function of the anatomic pulmonary valve in the systemic circulation. Circulation..

[7] Angeli E, Formigari R, Napoleone CP, Oppido G, Ragni L, Picchio FM (2010). Long-term coronary artery outcome after arterial switch operation for transposition of the great arteries. Eur J Cardiothorac Surg..

[8] Bonnet D, Bonhoeffer P, Piechaud JF, Aggoun Y, Sidi D, Planché C (1996). Long-term fate of the coronary arteries after the arterial switch operation in newborns with transposition of the great arteries. Heart..

[9] Hauser M, Bengel FM, Kühn A, Sauer U, Zylla S, Braun SL (2001). Myocardial blood flow and flow reserve after coronary reimplantation in patients after arterial switch and ross operation. Circulation..

[10] McMahon CJ, Ravekes WJ, Smith EO, Denfield SW, Pignatelli RH, Altman CA (2004). Risk factors for neo-aortic root enlargement and aortic regurgitation following arterial switch operation. Pediatr Cardiol..

[11] Vadi SK, Mehrotra S, Sood A, Parmar M, Dasagrandhi V, Kaur K, et al. Flip-flop right ventricle myocardial perfusion on stress-rest 99m Tc-MIBI myocardial perfusion scintigraphy: An indirect evidence for severe left ventricular coronary arterial disease? J Nucl Cardiol 2018. 10.1007/s12350-018-01565-z 30547296

[12] Higgins JP (2006). Increased right ventricular uptake on stress SPECT myocardial perfusion images in a patient with severe coronary artery disease. J Nucl Cardiol..

[13] Mannting F, Zabrodina YV, Dass C (1999). Significance of increased right ventricular uptake on 99mTc-Sestamibi SPECT in patients with coronary artery disease. J Nucl Med..

[14] Williams KA, Schneider CM, Chicago BS (1999). Increased stress right ventricular activity on dual isotope perfusion SPECT a sign of multivessel and/or left main coronary artery disease. J Am Coll Cardiol..

[15] Gaydos SS, Varga-Szemes A, Judd RN, Suranyi P, Gregg D (2017). Imaging in adult congenital heart disease. J Thorac Imaging..

[16] Hayes AM, Baker EJ, Kakadeker A, Parsons JM, Martin RP, Radley-Smith R (1994). Influence of anatomice correction for transposition (of the great arteries on myocardial perfusion: radionuclide imaging with technetium-99m 2-alethoyly isobutyl isonitrile. J Am Coll Cardiol..

[17] Weindling SN, Wernovsky G, Colan SD, Parker JA, Boutin C, Mone SM (1994). Myocardial perfusilon, function and exercise tolerance after the arterial switch operation. J Am Coll Cardiol..

[18] Huang MP, Liang CH, Zhao ZJ, Liu H, Li JL, Zhang JE (2011). Evaluation of image quality and radiation dose at prospective ECG-triggered axial 256-slice multi-detector CT in infants with congenital heart disease. Pediatr Radiol..

[19] Gimelli A, Achenbach S, Buechel RR, Edvardsen T, Francone M, Gaemperli O (2018). Strategies for radiation dose reduction in nuclear cardiology and cardiac computed tomography imaging: a report from the European Association of Cardiovascular Imaging (EACVI), the Cardiovascular Committee of European Association of Nuclear Medicine (EANM), and the European Society of Cardiovascular Radiology (ESCR) Imaging. Eur Heart J..

[20] Stout KK, Daniels CJ, Aboulhosn JA (2019). 2018 AHA/ACC guideline for the management of adults with congenital heart disease: A Report of the American College of Cardiology/American Heart Association Task Force on Clinical Practice Guidelines. Circulation..

[21] Sterrett LE, Schamberger MS, Ebenroth ES, Siddiqui AR, Hurwitz RA (2011). Myocardial perfusion and exercise capacity 12 years after arterial switch surgery for D-transposition of the great arteries. Pediatr Cardiol..

